# Risk Factors and Prognostic Effects of Refeeding Syndrome in Malnourished Elderly Patients During Nutritional Support

**DOI:** 10.1155/jnme/8521417

**Published:** 2026-08-25

**Authors:** Lin Zhang, Lu Meng, Han Sun, Zhihui Zhang, Yuexuan Liu, Shuhua Wu

**Affiliations:** ^1^ Department of Geriatrics, The Second Affiliated Hospital of Soochow University, Suzhou, Jiangsu, China, suda.edu.cn; ^2^ Department of General Medicine, The Second Affiliated Hospital of Soochow University, Suzhou, Jiangsu, China, suda.edu.cn; ^3^ Department of Emergency, Hulunbuir People’s Hospital, Hulunbuir, Inner Mongolia, China

**Keywords:** elderly, hypophosphatemia, malnutrition risk, nutritional intervention, prognosis, refeeding syndrome

## Abstract

**Background:**

Refeeding syndrome (RFS) is a life‐threatening metabolic complication arising when malnourished or catabolic patients resume nutrient intake, defined primarily by hypophosphatemia and concurrent electrolyte disturbances. Among malnourished elderly, RFS has an incidence of ∼50% and is linked to extremely high mortality.

**Methods:**

This retrospective case‐control study included 93 elderly patients (≥ 60 y) with a Nutrition Risk Screening 2002 (NRS 2002) score ≥ 3, admitted to two tertiary hospitals between August 2017 and August 2019. Patients were retrospectively assigned to the “RFS group” (*n* = 42, developed RFS within 1 week of admission) and a non‐RFS group (*n* = 51).

**Results:**

Independent RFS risk factors were low BMI (18.24 ± 1.66 vs. 22.06 ± 1.84 kg/m^2^), low serum albumin (29.97 ± 3.16 vs. 35.29 ± 4.33 g/L), and high D‐dimer (5.02 ± 1.87 vs. 2.34 ± 2.00 μg/mL; all *p* < 0.05). RFS patients had a significantly poorer prognosis: longer hospital stay (28.74 ± 9.02 vs. 16.84 ± 8.57 d), lower 28‐day (47.6% vs. 84.3%), and 90‐day (28.6% vs. 56.9%) survival rates, and higher organ dysfunction incidence (all *p* < 0.05). Within patients diagnosed with RFS, stepwise nutritional therapy contributed to better clinical outcomes. Among all enrolled high‐risk elderly individuals, nutritional regimens combined with phosphate, magnesium, potassium, vitamin B1 (thiamine) supplementation, and appropriate energy restriction effectively alleviated RFS severity and related complications. Subgroup analysis of RFS patients further confirmed that combined micronutrient supplementation shortened hospital stay and improved 90‐day survival.

**Conclusions:**

Universal micronutrient supplementation at admission combined with targeted energy restriction within the first 10–14 days of hospitalization reduces RFS risk among vulnerable elderly inpatients. Notably, multimicronutrient supplementation exerts a stronger protective effect against RFS than feeding route or initial energy regimen. Low BMI, hypoalbuminemia, and elevated D‐dimer serve as reliable predictors of RFS. Stepwise nutritional intervention with prompt electrolyte correction can shorten hospital stay and enhance 90‐day survival.

## 1. Introduction

First described in 1951 following the 20% mortality of starved World War II prisoners despite postrelease nutritional support, refeeding syndrome (RFS) is a life‐threatening metabolic complication of nutrient resumption in malnourished or catabolic patients [1]. Characterized primarily by hypophosphatemia and concurrent electrolyte disturbances, it manifests across systems—including circulatory (heart failure and arrhythmia), respiratory (dyspnea and pulmonary edema), nervous (muscle weakness and choking), and digestive (malabsorption and diarrhea), posing grave risks during nutritional therapy [2, 3]. As a metabolic complication during nutritional support therapy, RFS poses a potential threat to life.

Elderly patients are disproportionately vulnerable to RFS due to age‐related physiological decline, high comorbidity burdens, and depleted nutritional reserves. Key studies underscore its severity in this group: Lubart et al. [4] reported a 25% RFS incidence (1 in 4 nutritionally supported elderly patients) and acute mortality (22.5% within 1 week of diagnosis and 47.5% within 1 month of nasogastric feeding), with actual risk likely higher when preexisting acute conditions (e.g., infections and organ failure) are included. Bioletto et al. [5] further showed elevated medium‐term (≤ 6 months) mortality, refuting RFS as a “short‐term complication” and highlighting its long‐term impact via persistent metabolic dysfunction and reduced organ reserve. Overall, the two aforementioned studies clearly highlight the high incidence and mortality of RFS in older adults from both short‐term and medium‐term perspectives. This not only emphasizes the importance of RFS in the clinical management of elderly patients but also provides key references for future research directions, such as the construction of RFS risk prediction models, optimization of early intervention measures, and exploration of strategies to improve a long‐term prognosis. It holds significant guiding significance for enhancing the diagnosis, treatment quality, and survival prognosis of elderly RFS patients.

Current RFS risk assessment relies on the 2017 National Institute for Health and Care Excellence (NICE) guidelines [6], which cite low BMI, involuntary weight loss, alcohol history, and hypokalemia/hypophosphatemia/hypomagnesemia in the prereplenishment phase as high‐risk factors. However, these have poor sensitivity (30%–67%) and specificity (59%) [7, 8], leading to frequent missed diagnoses. Recent efforts to identify more sensitive markers (e.g., low prealbumin [9], low albumin [10], Nutritional Risk Screening [NRS] 2002 ≥ 3) [10, 11] remain inconsistent, with conflicting findings (e.g., failed to validate these factors) [12].

To address these gaps, this study conducted a retrospective case‐control analysis of data from elderly patients at risk of malnutrition. Baseline data, albumin, prealbumin, and other indicators at admission were collected, and their feeding methods, caloric intake, nutritional components, and prognostic status were recorded. The study aimed to retrospectively analyze the high‐risk factors for RFS in malnourished elderly patients, clarify the impact of RFS on the prognosis of elderly patients, and explore new high‐risk factors for RFS—with the goal of early detection and prevention of RFS and improving patient prognosis through stepwise nutritional therapy.

## 2. Materials and Methods

### 2.1. Study Population

The data of elderly patients at risk of malnutrition who met the inclusion and exclusion criteria and were admitted to the Emergency Critical Care Department of the Second Affiliated Hospital of Soochow University, Jiangsu province, transferred from this department to the Department of Geriatrics for further treatment, and admitted to the Emergency Intensive Care Unit of the People’s Hospital of Hulunbeier, Inner Mongolia province, between August 30, 2017, and August 30, 2019, were collected. All procedures involving human participants were conducted in accordance with the Declaration of Helsinki and approved by the Ethics Committee of the Second Affiliated Hospital of Soochow University (Approval No.: JD‐HG‐2017‐24). Informed consent was waived due to the retrospective nature of the study.

### 2.2. Inclusion Criteria

1. Aged ≥ 60 years.
2. NRS 2002 score ≥ 3 [13, 14].
3. Elderly patients in need of nutritional therapy. The NRS 2002 scoring system assesses three dimensions including current nutritional status, severity of underlying disease and age, and a total score ≥ 3 indicates a high risk of malnutrition.

### 2.3. Exclusion Criteria

1. Hospital stay < 7 days [15].
2. Severe renal failure: creatinine clearance rate (GFR) < 30 mL/min [16].
3. Severe liver failure: serum bilirubin ≥ 10 × upper limit of normal value or bilirubin increase ≥ 17.1 μmol/L per day, or bleeding tendency with prothrombin activity (PTA) ≤ 40%, or international normalized ratio (INR) ≥ 1.5 [17].
4. Hypophosphatemia caused by other reasons: undergoing hemodialysis treatment, renal tubular acidosis, Fanconi syndrome, hyperparathyroidism [18], and undergoing treatment for hyperphosphatemia.
5. Patients with baseline hypophosphatemia at admission (serum phosphate < 0.6 mmol/L) to exclude individuals with preexisting hypophosphatemia unrelated to nutritional refeeding.

### 2.4. Diagnostic Criteria

There is currently no unified diagnostic standard for RFS. This study adopted the standard proposed by Friedli et al. [19]: All enrolled patients received baseline blood electrolyte tests immediately after admission. Patients with preexisting hypophosphatemia were excluded according to the exclusion criteria. After baseline assessment, patients received the nutritional support deemed most clinically appropriate by the attending physician and registered dietitian (RD). RFS assessment was uniformly performed 72 h after the initiation of nutritional therapy.

Patients who met any of the following conditions were assigned to the study group (RFS group): serum phosphate decreased by more than 30% compared with admission baseline level, or serum phosphate < 0.6 mmol/L, or two or more other electrolytes decreased below the normal range within 72 h of nutritional therapy. Notably, diagnosis and group classification were performed exclusively according to laboratory values.

### 2.5. Experimental Methods

#### 2.5.1. Grouping

1. According to the diagnostic criteria for RFS, elderly patients with RFS were set as the study group, and elderly patients without RFS were set as the control group.
2. We categorized the nutritional regimens into three dimensions for subgroup analysis: feeding regimen, nutritional component formulation, and feeding energy supply regimen. In this retrospective study, nutritional regimens (total energy supply) were determined by attending physicians based on patients’ nutritional risk severity, organ function status, overall physical condition, and clinical routine treatment principles in real clinical practice. The feeding regimen subgroup encompassed three methods: (1) Enteral nutrition: This includes oral feeding and nasogastric tube feeding. Alert patients with intact swallowing function received oral feeding. Nasogastric feeding is indicated when oral nutrition does not meet the patient’s nutritional requirements, such as in patients with coma or dysphagia. (2) Parenteral nutrition: Parenteral nutrition is indicated for patients who cannot meet their nutritional requirements through oral and/or enteral nutrition, who do not tolerate enteral nutrition, or who have contraindications to it (such as gastrointestinal (GI) bleeding, severe digestive and absorption disorders, intractable vomiting, intestinal obstruction, and stress status). (3) Combined nutrition: A combination of enteral and parenteral nutrition was adopted for patients with partial GI function who could not meet total nutritional demands via single enteral feeding.
 We further stratified patients using three independent, hierarchically organized nutritional regimen variables, analyzed separately and in cross‐combination:
  1. Feeding regimen (3 mutually exclusive categories): enteral nutrition, parenteral nutrition, and combined enteral‐parenteral nutrition.
  2. Prophylactic multimicronutrient supplementation status (binary): patients who received routine concurrent phosphate, magnesium, potassium, and thiamine supplementation at nutritional support initiation, and patients who did not receive this standardized prophylactic bundle. The standardized prophylactic bundle used fixed daily doses for the initial 10–14 days of nutritional support, with route selection determined by GI tolerance and feeding regimen:
    • Thiamine (vitamin B1): 200–300 mg daily; intravenous (IV) infusion for patients unable to take oral intake, oral tablets for those with intact swallowing/enteral access
    • Phosphate: 0.3–0.6 mmol/kg daily; oral phosphate salts for mild baseline risk, IV disodium hydrogen phosphate for severe malnutrition or poor oral tolerance
    • Magnesium: 0.2 mmol/kg IV daily or 0.4 mmol/kg oral daily via magnesium sulfate preparations
    • Potassium: 2–4 mmol/kg daily; oral potassium chloride as first‐line, IV potassium reserved for baseline serum potassium < 3.5 mmol/L
   No patients received isolated phosphate prophylaxis alone; unsupplemented patients only received delayed, ad hoc single‐electrolyte correction if postfeeding laboratory hypophosphatemia, hypomagnesemia, or hypokalemia was detected.
  3. Energy dosing regimen (binary): standard energy (30–40 kcal/kg/day) or restricted energy (10–29 kcal/kg/day) for the initial 1‐2 weeks of refeeding.
 Decision to provide prophylactic multimicronutrient supplementation was determined by attending clinicians based on admission NRS 2002 score, baseline BMI, serum albumin levels, and preexisting electrolyte trends, per hospital nutritional support protocols: patients with high malnutrition risk (NRS 2002 ≥ 4, BMI < 18.5 kg/m^2^, albumin < 30 g/L) routinely received upfront phosphate, magnesium, potassium, and thiamine; patients who, regardless of their NRS 2002 score, were deemed to have a lower nutritional risk and normal admission electrolytes did not receive standardized prophylactic supplementation. Energy dosing was individualized by clinicians based on organ function, baseline malnutrition severity, and perceived RFS risk, independent of supplementation decisions.
3. Subgroup analysis was performed: Within the RFS case cohort, we retrospectively stratified patients into two analytical subgroups based on the real‐world nutritional regimens they received during clinical care: patients managed with stepwise nutritional therapy and patients receiving fixed standard energy feeding without staged calorie escalation. The stepwise nutritional therapy protocol initiates feeding with restricted energy, then gradually increases energy intake over 10–14 days to reach standard energy levels.

Per institutional standard clinical guidelines, patients presenting with obvious admission electrolyte disturbances or severe malnutrition were recommended to receive routine supplementation of phosphate, magnesium, potassium, and thiamine, whereas patients with normal baseline electrolytes and perceived low RFS risk were not routinely prescribed these micronutrients. However, this guideline was not fully uniformly implemented in real‐world clinical practice. A subset of patients who met the supplementation indications failed to receive targeted supplements due to clinical constraints, including spontaneous correction of mild electrolyte abnormalities within 24 h, fluid overload contraindications to IV repletion, and delayed enteral feeding access. Meanwhile, a small number of low‐risk patients without formal supplementation indications received discretionary micronutrient repletion at attending physicians’ clinical judgment. Supporting stratified quantitative analyses (Supporting Table 3) were added to quantify: (1) the proportion of eligible patients with admission electrolyte disorders or severe malnutrition who actually received supplements and (2) the distribution of baseline risk status among all supplemented patients (high‐risk eligible patients versus low‐risk patients without standard indications). All outcome comparisons related to micronutrient supplementation were further adjusted for baseline supplementation eligibility in additional logistic regression models to address confounding from inconsistent guideline adherence.

#### 2.5.2. Nutritional Therapy Regimens

During hospitalization, patients received two related but distinct physician‐directed nutritional strategies, prescribed at the attending clinician’s discretion according to each patient’s estimated RFS risk. Restricted energy refers to fixed low caloric supply (10–29 kcal/kg/day); stepwise nutritional therapy is a staged protocol that begins with restricted energy and gradually escalates energy intake to the standard level within 10–14 days. Within the study group, the patients were divided into the stepwise nutritional therapy group and the standard energy group [20], and the specific methods were as follows [21].

For stepwise nutritional therapy group, the initial energy intake was 10 kcal/kg/day, and the energy intake was gradually increased to 20 kcal/kg/day within 4–7 days. The total nutritional intake was adjusted dynamically according to the GI tolerance of each patient, including GI motility, abdominal pain, nausea, vomiting, diarrhea, and other adverse reactions related to feeding. The full energy requirement of 30–40 kcal/kg/day was reached within 10–14 days. The energy composition was 40%–60% carbohydrates, 30%–40% fats, and 15%–20% proteins.

It is critical to distinguish two independent electrolyte management strategies applied in this cohort: (1) prophylactic bundled micronutrient supplementation initiated at admission (phosphate, magnesium, and potassium plus thiamine, detailed in Section 2.5.1), a preventive intervention given daily for the initial 1‐2 weeks of nutritional support to patients with severe malnutrition or baseline electrolyte disturbances and (2) targeted rescue electrolyte correction for patients who developed new‐onset electrolyte deficits after nutritional initiation. For rescue electrolyte correction of postfeeding hypophosphatemia, patients with moderate to severe hypophosphatemia (≤ 0.6 mmol/L) received stratified phosphate replacement. For critically ill patients with severe hypophosphatemia and poor oral intake, IV disodium hydrogen phosphate was administered (0.9% sodium chloride/5% glucose injection + 2 mL disodium hydrogen phosphate, 1.0 mol/L). To reduce the risk of insulin surge and subsequent intracellular phosphorus shift, 0.9% normal saline was preferentially used as the diluent instead of 5% glucose (D5); 5% glucose solution was only applied for patients with concurrent hypoglycemia and used at the minimum required volume. For patients with mild hypophosphatemia or good oral tolerance, oral phosphorus supplements were adopted as the first‐line therapy to avoid the adverse effects of IV dextrose. Patients with severe hypomagnesemia (< 0.5 mmol/L) received IV infusion of magnesium sulfate (5% glucose + 10 mL magnesium sulfate, 2 mmol/mL). For patients with serum potassium lower than 3.5 mmol/L, oral potassium supplementation was the first choice, and IV potassium supplementation was administered if necessary. All rescue electrolyte repletion was discontinued once serum phosphate, magnesium, and potassium levels returned to normal reference ranges.

### 2.6. Recording of Experimental Indicators

The general situation and clinical data of the enrolled patients were recorded as following: age, gender, BMI, muscle atrophy, fat loss (by body composition analyzer), weight loss in the past 3 months, history of geriatric comorbidities, tumor history, alcohol consumption history, chronic disease status, history of medications known to disrupt electrolyte balance or worsen malnutrition (including diuretics, corticosteroids, antacids, chemotherapy agents, and proton pump inhibitors), albumin, prealbumin, creatinine, urea nitrogen, routine blood test, blood glucose, coagulation indicators, c‐reactive protein, procalcitonin, APACHE II score, SOFA score, NRS 2002 score, feeding regimen, nutritional components, feeding energy, mechanical ventilation/high‐flow nasal cannula (HFNC)/noninvasive ventilation (NIV), energy intake per hour at admission, and total energy intake within 24 h before admission. In addition, prognostic indicators were also recorded, including the length of hospital stay, duration of mechanical ventilation, number of deaths, number of patients with adverse clinical outcomes including failure to recover, deterioration or death, number of improved cases, 28‐day survival rate, 90‐day survival rate [20], new infections, organ damage, and combined treatment status. All baseline laboratory indicators were collected within 24 h after admission. Dynamic electrolyte data within 72 h after nutritional initiation were recorded strictly for RFS diagnosis and grouping. All enrolled patients were jointly managed by attending physicians and RDs. RDs conducted routine nutritional assessment and follow‐up at least once per day during hospitalization to monitor feeding tolerance and adjust regimens timely. Nutritional escalation decisions were mainly based on objective GI tolerance indicators rather than patients’ subjective feelings. Patients with relieved abdominal discomfort, improved GI motility, and no feeding‐related adverse reactions were permitted to gradually increase nutritional intake.

### 2.7. Statistical Analysis

The collected patient data were entered into a computer database, and SAS 9.4 software was used for data statistics and analysis. Measurement data were expressed as mean ± standard deviation (*x* ± *s*), and count data were expressed as the number of cases or rate (%). The *t*‐test was used for comparing the two groups and before and after treatment, and the *χ* ^2^ test was used for comparing groups. Univariate analysis was performed on the collected data to screen out indicators with statistical significance, and binary logistic regression analysis was performed. Besides the primary univariate and multivariate logistic regression models, an additional adjusted logistic regression model was constructed incorporating baseline supplementation eligibility as a covariate to eliminate confounding bias stemming from incomplete adherence to institutional micronutrient supplementation guidelines. A *p* value < 0.05 was considered statistically significant.

## 3. Results

### 3.1. Baseline Characteristics of the Study Population

According to the inclusion and exclusion criteria, a total of 93 elderly patients with malnutrition who met the study criteria were included, comprising 37 females and 56 males, with a mean age of 76.40 ± 8.99 years. According to the diagnostic criteria, 42 cases met the criteria for RFS and thus were identified as the study group, while the remaining 51 cases comprised the control group. Notably, both the diagnosis and group classification in this study were determined solely based on laboratory parameters.

Baseline characteristics of the study population are presented in Table 1. The BMI of the study group (18.24 ± 1.66 kg/m^2^) was significantly lower than that of the control group (22.06 ± 1.84 kg/m^2^), and the difference was statistically significant (*p* = 0.0001). There were no statistically significant differences in age, gender, alcohol consumption history, history of geriatric comorbidities, muscle atrophy, fat loss, chronic disease status, APACHE II score, SOFA score, NRS 2002 score, and assisted ventilation between the two groups (*p* > 0.05). Notably, no enrolled patients had baseline hypophosphatemia at admission, as such cases were excluded in accordance with our preset exclusion criteria. All electrolyte abnormalities included in subsequent analysis occurred after the initiation of nutritional therapy. Clinical systemic manifestations exclusively occurred in patients who developed RFS: 17 cases (40.5%) presented respiratory symptoms including respiratory failure, tachypnea, and respiratory weakness, and 15 cases (35.7%) developed heart failure or arrhythmia, with zero cases observed in the non‐RFS group (Table 1).

**TABLE 1:** Baseline characteristics of the study population (*x* ± *s*, *n*).

Parameters	Study group (*n* = 42)	Control group (*n* = 51)	*χ* ^2^/*t* value	*p* value
Age (years)	75.26 ± 7.78	77.33 ± 9.85	1.11	0.27
Gender (male/female)	27/15	29/22	0.53	0.47
Alcohol consumption history (*n*)	32	36	0.37	0.54
Tumor history (*n*)	20	24	0.99	0.32
Hypertension history (*n*)	22	25	0.10	0.75
Diabetes history (*n*)	11	10	0.57	0.45
BMI (kg/m^2^)	18.24 ± 1.66	22.06 ± 1.84	10.42	< 0.0001^∗^
BMI < 18 kg/m^2^ (*n*)	20	7	12.84	0.0003^∗^
Calf circumference (cm)	2.11 ± 0.19	2.11 ± 0.22	−0.01	0.99
Fat loss (%)	2.14 ± 0.18	2.14 ± 0.17	−0.09	0.93
Phosphorus (mmol/L)	1.00 ± 0.28	1.04 ± 0.22	0.61	0.54
Magnesium (mmol/L)	0.81 ± 0.10	0.82 ± 0.11	0.11	0.91
Potassium (mmol/L)	3.89 ± 0.32	3.87 ± 0.37	−0.27	0.79
Immunocompromise (*n*)	20	19	1.02	0.31
Respiratory diseases (*n*)	21	23	0.22	0.64
Cardiovascular diseases (*n*)	19	24	0.03	0.86
Insulin‐dependent diabetes mellitus (*n*)	6	3	1.86	0.17
Liver cirrhosis (*n*)	9	10	0.05	0.83
Chronic dialysis (*n*)	4	7	0.39	0.53
APACHE II score	14.83 ± 4.88	17.00 ± 5.76	1.93	0.06
SOFA score	7.33 ± 3.08	7.96 ± 3.99	0.83	0.41
NRS 2002 score	4.45 ± 1.04	4.27 ± 1.13	−0.78	0.44
HFNC/NIV (*n*)	7	9	0.02	0.90
Mechanical ventilation (*n*)	9	6	1.59	0.21
Respiratory system manifestations (respiratory failure, tachypnea, respiratory weakness), *n* (%)	17 (40.5)	0 (0)	43.21	< 0.0001^∗^
Circulatory system manifestations (heart failure, arrhythmia), *n* (%)	15 (35.7)	0 (0)	36.45	< 0.0001^∗^

Abbreviations: HFNC, high‐flow nasal cannula; NIV, noninvasive ventilation.

^∗^ *p* < 0.05 indicates a statistically significant difference compared with the study group and the control group.

### 3.2. Analysis of Risk Factors for RFS in Elderly Patients During Nutritional Support Therapy

#### 3.2.1. Classification of Main Risk Factors Analysis

The full cross‐distribution of patients across feeding route, multimicronutrient supplementation status, and energy dosing subgroups is summarized in Supporting Table 1 to clarify sample sizes across all nutritional intervention exposure strata.

The results showed no statistically significant intergroup difference in feeding delivery modalities (enteral, parenteral, or combined nutrition) between the study and control groups (*p* > 0.05). Calorie intake categories (restricted vs. standard energy) were analyzed separately below. Within the parenteral nutrition group, according to the different infusion methods, the patients were divided into the PICC group, medium‐ and long‐term central catheter group, infusion port group, and deep vein group, and there was no statistically significant difference among the groups (*p* > 0.05). According to the intake of different nutritional components including micronutrients, the number of RFS cases was fewer in patients receiving the combined supplementation of phosphate, magnesium, potassium, and thiamine (*p* < 0.05). Notably, this supplementation bundle was preferentially prescribed to patients with severe malnutrition or admission electrolyte disturbances who carried inherently elevated RFS risk; detailed data on guideline adherence and risk stratification of supplemented patients are presented in Supporting Table 2. An additional adjusted logistic regression model (Supporting Table 3), which controlled for baseline eligibility for supplementation, further verified that this micronutrient bundle was independently protective against RFS despite the higher baseline risk of most recipients. According to the intake of different nutritional components including micronutrients, the number of RFS cases was fewer in the group receiving phosphate, magnesium, potassium, and vitamin B1 (*p* < 0.05). According to the different feeding energy regimens, the number of RFS cases was fewer in the restricted energy group (10–29 kcal/kg/day, *p* < 0.05). This restricted energy strategy was only applied in the acute early stage (the first 1‐2 weeks) of nutritional support. To avoid worsening malnutrition, we maintained adequate protein supply throughout the whole treatment and gradually escalated total energy intake following the stepwise nutritional protocol afterward [22]. The specific values are shown in Table 2, and the subgroup analysis of parenteral administration is shown in Supporting Table 4. It classifies parenteral nutrition patients into four subgroups: PICC group, medium‐ and long‐term central catheter group, infusion port group, and deep vein group, and compares the incidence of RFS among these subgroups. The results show that there is no statistically significant difference in RFS incidence between the subgroups (*p* > 0.05), indicating that the choice of the parenteral nutrition administration method does not affect the risk of RFS. All parenteral nutrition formulations had identical macronutrient, micronutrient, and caloric compositions across groups. The total energy of parenteral nutrition was adjusted according to the unified standard energy or restricted energy criteria defined above, while the basic formula remained unchanged. For enteral nutrition, standard commercial enteral formulas were used, with total energy matching the grouping criteria as well.

**TABLE 2:** Comparison of refeeding syndrome incidence across different nutritional support regimens.

Category	Type	Study group (*n* = 42)	Control group (*n* = 51)	*χ* ^2^/*t* value	*p* value
Feeding regimen	Enteral nutrition	10	13	0.14	0.93
	Parenteral nutrition	19	24		
	Combined nutrition	13	14		

Feeding components	With phosphate, magnesium, potassium, and vitamin B1	12	28	6.51	0.01^∗^
	Without phosphate, magnesium, potassium, and vitamin B1	30	23		

Feeding energy	Standard energy	30	19	10.79	0.001^∗^
	Restricted energy	12	32		

^∗^ *p* < 0.05 indicates a statistically significant difference.

To further clarify the prognostic benefit of combined phosphate, magnesium, potassium, and thiamine supplementation among patients who already developed RFS, we performed a post hoc stratified analysis within the RFS cohort (*n* = 42). Patients were split into two subgroups: those receiving full multimicronutrient supplementation at nutritional initiation (*n* = 12) and those without routine combined supplementation (*n* = 30). Supporting Table 5 summarizes all comparative prognostic endpoints between the two RFS subgroups. As presented in Supporting Table 5, RFS patients receiving complete micronutrient supplementation exhibited significantly shorter hospital stays, lower in‐hospital mortality, higher 28‐day and 90‐day survival rates, and reduced incidence of respiratory and cardiac insufficiency, as well as shorter duration of mechanical ventilation, pressure ulcer care, persistent hypoalbuminemia, and systemic antibiotic treatment (all *p* < 0.05). No significant intergroup differences were observed in liver, renal, coagulation, and multiple organ dysfunction rates (*p* > 0.05).

For control group patients who received supporting electrolytes, interventions were initiated strictly after baseline blood tests and before the 72‐h RFS evaluation window. Standardized dynamic electrolyte monitoring was performed for all participants within 72 h of nutritional initiation, so early electrolyte supplementation did not mask the diagnosis of new‐onset RFS.

#### 3.2.2. Univariate Analysis of Risk Factors

The results showed that the prealbumin of the study group (0.15 ± 0.02 g/L) was lower than that of the control group (0.25 ± 0.70 g/L); the albumin of the study group (29.97 ± 3.16 g/L) was lower than that of the control group (35.29 ± 4.33 g/L); the D‐dimer of the study group (5.02 ± 1.87 μg/mL) was higher than that of the control group (2.34 ± 2.00 μg/mL), all with *p* < 0.05. There were no statistically significant differences in the blood cell count, fibrin, inflammatory indicators, blood glucose, creatinine, urea nitrogen, and other items between the study group and the control group before nutritional therapy (*p* > 0.05), as shown in Table 3.

**TABLE 3:** Univariate analysis of risk factors for RFS during nutritional support (*x* ± *s*, *n*).

Parameters	Study group	Control group	*χ* ^2^/*t* value	*p* value
Prealbumin (g/L)	0.15 ± 0.02	0.25 ± 0.70	23.16	< 0.0001^∗^
Albumin (g/L)	29.97 ± 3.16	35.29 ± 4.33	6.63	< 0.0001^∗^
Creatinine (μmol/L)	67.30 ± 15.85	67.74 ± 15.10	0.13	0.89
Urea nitrogen (mmol/L)	10.06 ± 1.81	9.83 ± 2.06	0.54	0.59
White blood cells (10^9^/L)	11.33 ± 3.91	11.13 ± 3.03	1.20	0.23
Hemoglobin (g/L)	115.60 ± 19.35	116.10 ± 18.35	0.11	0.92
Platelets (10^9^/L)	195.70 ± 41.42	194.70 ± 40.56	−0.12	0.91
Neutrophil count (10^9^/L)	8.64 ± 1.44	9.16 ± 1.24	1.88	0.06
Lymphocyte count (10^9^/L)	0.97 ± 0.29	0.94 ± 0.31	−0.50	0.62
Neutrophil/lymphocyte ratio	9.92 ± 3.84	12.45 ± 12.71	1.35	0.18
Platelet/lymphocyte ratio	224.60 ± 101.30	257.20 ± 236.90	0.89	0.38
Fibrinogen (g/L)	4.04 ± 1.37	4.21 ± 1.30	0.60	0.55
D‐dimer (μg/mL)	5.02 ± 1.87	2.34 ± 2.00	−6.63	< 0.0001^∗^
C‐reactive protein (mg/L)	4.13 ± 0.98	3.89 ± 1.39	−0.96	0.34
Procalcitonin (ng/mL)	0.60 ± 0.11	0.58 ± 0.09	−0.74	0.46
Weight loss in the past 3 months (kg)	5.52 ± 1.25	5.31 ± 1.38	−0.76	0.45
Energy intake per hour at admission (kcal/h)	43.32 ± 8.95	41.95 ± 8.09	−0.78	0.44
Total energy intake within 24 h before admission (kcal)	469.70 ± 38.79	455.70 ± 32.28	−1.90	0.06
Maximum blood glucose within 24 h (mmol/L)	8.14 ± 1.82	8.45 ± 1.79	0.82	0.41
Minimum blood glucose within 24 h (mmol/L)	3.98 ± 1.97	4.13 ± 1.98	0.37	0.72
Long‐term or high‐dose corticosteroids	7	9	0.02	0.90
Distal loop diuretics (taken within 24 h)	13	9	2.26	0.13
History of high alcohol intake	15	12	1.66	0.20

^∗^ *p* < 0.05 indicates a statistically significant difference compared with the study group and the control group.

#### 3.2.3. Multivariate Logistic Regression Analysis of Risk Factors for RFS During Nutritional Support

The meaningful independent variables screened out from the univariate comparison results, including BMI, serum albumin, prealbumin, and D‐dimer, were analyzed by binary logistic regression. The results showed that the OR value of BMI was < 1, indicating that in elderly malnourished patients with low BMI, for each 1‐unit increase in BMI (low BMI was defined as BMI < 18.5 kg/m^2^), the risk of RFS decreased to 0.28 times the original level. The OR value of albumin was < 1. For each 1 g/L rise in serum albumin, the risk of RFS decreased to 0.72 times the original level. The OR value of D‐dimer was > 1. For each 1 μg/mL rise in D‐dimer, the risk of RFS increased to 2.2 times the original level. Prealbumin was not suitable for entering the regression analysis model and may be a potential risk factor. The above results suggest low BMI, low albumin, and high D‐dimer levels before nutritional therapy are risk factors for RFS. The specific values are shown in Supporting Table 6.

Prealbumin, although lower in the RFS group in univariate analysis, is excluded from the regression model due to confounding factors (e.g., liver dysfunction and inflammation), suggesting it may be a potential but nonindependent risk factor. This table provides direct statistical evidence for the identification of RFS high‐risk groups in clinical practice.

To address variable compliance with the supplementation protocol, we generated Supporting Table 7 to quantify the proportion of eligible patients receiving supplements and baseline risk distribution of all supplemented individuals. A supporting adjusted logistic regression model (Supporting Table 8) further isolated the independent protective effect of phosphate, magnesium, potassium, and thiamine supplementation after controlling for baseline clinical indications for repletion.

### 3.3. Comparative Analysis of Prognostic Indicators in Elderly Patients During Nutritional Support Therapy

#### 3.3.1. Prognostic Indicators

The average length of hospital stay was 28.74 ± 9.02 in the study group vs. 16.84 ± 8.57 in the control group, *p* < 0.05. The number of deaths and nonrecovered cases in the study group was higher than that in the control group, *p* < 0.05. The number of improved cases, 28‐day survival rate, and 90‐day survival rate in the study group were lower than those in the control group, *p* < 0.05. In the study group, the number of patients with respiratory and cardiac insufficiency and the number of days of mechanical ventilation, pressure ulcer treatment, hypoalbuminemia, and systemic antibiotic use were higher than that in the control group, *p* < 0.05, as shown in Supporting Table 9. It systematically compares the differences in prognostic indicators between the RFS group and the non‐RFS group, including the average length of hospital stay, number of deaths, number of nonrecovered cases, number of improved cases, 28‐day survival rate, 90‐day survival rate, incidence of organ dysfunction (respiratory insufficiency and cardiac insufficiency), and duration of combined treatment (mechanical ventilation and pressure ulcer treatment).

#### 3.3.2. Impact of Hypophosphatemia on Prognosis in the Study Group

Within the study group, according to the blood phosphate level at admission, the patients with a blood phosphate level of 0.81–1.55 mmol/L were included in the normal blood phosphate group, and those with a blood phosphate level lower than 0.81 mmol/L were included in the hypophosphatemia group. The results showed that the length of hospital stay, number of deaths, and number of nonrecovered cases in the hypophosphatemia group were higher than those in the normal blood phosphate group. The number of improved cases, 28‐day survival rate, and 90‐day survival rate were lower than those in the normal blood phosphate group, with statistically significant differences (*p* < 0.05). The number of patients with respiratory and cardiac insufficiency in the hypophosphatemia group was higher than that in the normal blood phosphate group. The number of days of mechanical ventilation, number of days of pressure ulcer treatment, number of days of hypoalbuminemia, and number of days of systemic antibiotic use in the hypophosphatemia group were higher than those in the control group, *p* < 0.05, as shown in Table 4.

**TABLE 4:** Impact of hypophosphatemia on prognosis in the study group (*x* ± *s*, *n*%).

Parameters	Normal blood phosphate group (*n* = 20)	Hypophosphatemia group (*n* = 22)	*χ* ^2^/*t* value	*p* value
Average length of hospital stays (days)	28.00 ± 8.35	30.38 ± 10.53	−0.79	0.44^∗^
Number of deaths (*n*)	5	12	19.61	< 0.0001^∗^
Number of nonrecovered (*n*)	3	7	12.17	0.0005^∗^
Number of improved (*n*)	12	3	6.48	0.01^∗^
28‐day survival rate (%)	86.21	53.85	5.18	0.02^∗^
90‐day survival rate (%)	51.72	15.38	4.92	0.03^∗^
New infections (*n*)	9	7	1.98	0.16

*Organ dysfunction*				
‐Respiratory insufficiency (*n*)	5	9	10.92	0.001^∗^
‐Liver insufficiency (*n*)	3	4	2.70	0.10
‐Cardiac insufficiency (*n*)	3	8	12.17	0.0005^∗^
‐Coagulation disorders (*n*)	6	5	0.87	0.35
‐Renal insufficiency (*n*)	15	6	0.11	0.74
‐Multiple organ damage (*n*)	6	5	1.47	0.23

*Combined treatment*				
‐Number of days of mechanical ventilation (days)	8.23 ± 4.34	9.72 ± 3.69	1.15	0.26^∗^
‐Number of days of pressure ulcer treatment (days)	10.45 ± 1.43	10.62 ± 1.39	−0.35	0.73^∗^
‐Number of days of hypoalbuminemia (days)	17.77 ± 4.80	17.93 ± 5.26	0.09	0.93^∗^
‐Number of days of systemic antibiotic use (days)	13.76 ± 2.67	14.23 ± 2.65	−0.53	0.60^∗^

^∗^ *p* < 0.05 indicates a statistically significant difference compared to the normal blood phosphate and hypophosphatemia groups.

#### 3.3.3. Prognosis of Different Nutritional Regimens in the Study Group

The results showed that within the study group, the length of hospital stay in the stepwise nutritional therapy group was (18.39 ± 8.53) days, which was significantly shorter than that in the standard energy group (28.48 ± 7.26) days, *p* < 0.05. The number of deaths and nonrecovered cases in the stepwise nutritional therapy group was lower than that in the standard energy group, and the number of improved cases, 28‐day survival rate, and 90‐day survival rate was higher than that in the standard energy group, *p* < 0.05. The number of patients with respiratory and cardiac failure in the stepwise nutritional therapy group was lower than that in the standard energy group. The number of days of mechanical ventilation, number of days of pressure ulcer treatment, number of days of hypoalbuminemia, and number of days of systemic antibiotic use in the stepwise nutritional therapy group were lower than that in the standard energy group, *p* < 0.05, as shown in Table 5.

**TABLE 5:** Comparative analysis of clinical prognosis in RFS patients by early caloric energy intake (*x* ± *s*, *n*%).

Parameters	Standard energy group (*n* = 24)	Stepwise nutritional therapy group (*n* = 18)	*χ* ^2^/*t* value	*p* value
Average length of hospital stays (days)	28.48 ± 7.26	18.39 ± 8.53	2.46	0.03^∗^
Number of deaths (*n*)	12	5	4.84	0.03^∗^
Number of nonrecovered (*n*)	9	1	8.40	0.0038^∗^
Number of improved (*n*)	3	12	6.11	0.01^∗^
28‐day survival rate (%)	28.57	66.67	6.11	0.01^∗^
90‐day survival rate (%)	14.29	42.86	4.20	0.04^∗^
New infections (*n*)	5	4	0.14	0.71

*Organ dysfunction*				
‐Respiratory insufficiency (*n*)	10	3	4.61	0.03^∗^
‐Liver insufficiency (*n*)	3	4	0.17	0.68
‐Cardiac insufficiency (*n*)	15	6	7.71	0.01^∗^
‐Coagulation disorders (*n*)	2	3	0.13	0.72
‐Renal insufficiency (*n*)	8	6	0.43	0.51
‐Multiple organ damage (*n*)	9	7	0.40	0.53

*Combined treatment*				
‐Number of days of mechanical ventilation (days)	12.21 ± 6.71	5.47 ± 3.37	1.60	0.02^∗^
‐Number of days of pressure ulcer treatment (days)	14.89 ± 3.69	8.72 ± 1.63	2.55	0.01^∗^
‐Number of days of hypoalbuminemia (days)	19.29 ± 5.21	10.26 ± 3.89	0.69	0.00^∗^
‐Number of days of systemic antibiotic use (days)	15.57 ± 3.33	10.21 ± 2.27	0.77	0.00^∗^

^∗^ *p* < 0.05 indicates a statistically significant difference compared with the standard energy group and stepwise nutritional therapy group.

### 3.4. Changes in Electrolyte Levels Before and After Nutritional Therapy in the Study Group and the Control Group

1. Phosphate, magnesium, and potassium concentrations on Day 3 of nutrition were significantly lower relative to baseline in the study group, *p* < 0.05, whereas no meaningful electrolyte shifts were observed in the control group, *p* > 0.05. Full details on the number of patients who received standard prophylactic phosphate–magnesium–potassium–thiamine supplementation versus those who did not are provided in Supporting Table 7
2. In the study group, there was no statistically significant difference in the levels of phosphate, magnesium, and potassium on the 7th day after nutritional therapy compared with those before nutritional therapy, *p* > 0.05. In the control group, there was no statistically significant difference in the levels of phosphate, magnesium, and potassium on the 7th day after nutritional therapy compared with those before nutritional therapy, *p* > 0.05, as shown in Supporting Table 10. It records the dynamic changes in serum phosphate, magnesium, and potassium levels in the RFS group and non‐RFS group before nutritional therapy, on the 3rd day, and on the 7th day after therapy. The results show that in the RFS group, phosphate, magnesium, and potassium levels on the 3rd day after therapy are significantly lower than those before therapy, *p* < 0.05, but return to baseline levels on the 7th day; in the non‐RFS group, there is no significant change in electrolyte levels during the same period, *p* > 0.05. These data not only confirm the timeline of RFS‐related electrolyte disturbances (onset within 72–96 h after feeding) but also verify the effectiveness of early electrolyte monitoring and intervention in correcting electrolyte imbalances.

### 3.5. Analysis of Main Diseases at Admission in the Study Group

In this study, there were 42 cases in the study group, including 20 cases of tumors, accounting for 48%, 7 cases of pneumonia, accounting for 17%, and 6 cases of inflammatory diseases of the digestive system, accounting for 14%. It can be observed that the diseases complicated by RFS are mainly tumors and inflammatory diseases. See Supporting Figure 1 for details.

## 4. Discussion

RFS remains a life‐threatening metabolic complication in malnourished elderly patients undergoing nutritional support, yet its clinical recognition and management remain suboptimal. This retrospective case‐control study investigated RFS risk factors, prognostic impacts, and the efficacy of targeted nutritional interventions in 93 elderly patients (≥ 60 years) with nutritional risk (NRS 2002 ≥ 3) at the time of inpatient hospital admission. The findings align with and extend existing evidence, while addressing critical gaps in understanding RFS in this vulnerable population.

### 4.1. Key Risk Factors for RFS in Malnourished Elderly Patients

Clarifying and refining current risk stratification frameworks (e.g., NICE guidelines [6]), this study identified low BMI, low serum albumin, and high D‐dimer as independent risk factors for RFS prediction in this elderly population. Nearly half (47.6%) of all RFS patients presented with a BMI < 18 kg/m^2^, compared with only 13.7% of non‐RFS control patients. This aligns with Friedli et al.’ s [10] systematic review, which linked low BMI to impaired metabolic reserve, a critical precursor to RFS. As per ESPEN criteria [25, 26], low BMI reflects severe protein‐energy malnutrition, which disrupts cellular energy stores (e.g., ATP) and electrolyte homeostasis. When nutrition is resumed, the shift from catabolism to anabolism exacerbates electrolyte depletion (e.g., phosphate, magnesium, and potassium), triggering RFS. Consistent with this pattern, substantially more RFS patients had serum albumin below 30 g/L at admission relative to controls, and mean albumin concentrations were 15% lower among those who developed RFS. Albumin, a liver‐synthesized protein, is a reliable marker of chronic malnutrition and vascular integrity. Low albumin reduces plasma colloid osmotic pressure, promoting fluid shifts and electrolyte imbalances—key drivers of RFS‐related complications (e.g., edema, heart failure) [27–30]. Notably, prealbumin (a more sensitive but unstable nutritional marker) was lower in the RFS group. Still, it was excluded from the regression model, likely due to confounders (e.g., liver dysfunction and inflammation) common in elderly patients with comorbidities [17]. D‐dimer concentrations were markedly elevated in the RFS cohort, with far more RFS patients presenting clinically elevated D‐dimer levels upon admission versus controls, a disparity consistent with the study’s high proportion of participants with active malignancy or severe infection. Tumor cells secrete tissue factors that disrupt the coagulation‐fibrinolysis balance [31], while severe infection (e.g., pneumonia) increases D‐dimer via inflammation‐driven coagulation activation [32]. Both pathways may exacerbate metabolic stress, priming patients for RFS when nutrition is restarted.

This study powerfully demonstrates that provision of a standardized phosphate, magnesium, potassium, and vitamin B1/thiamine micronutrient bundle markedly reduces the risk of RFS even in high‐risk patients. Across the full cohort, prophylactic multimicronutrient supplementation significantly reduced the overall likelihood of developing RFS, serving as an effective preventive intervention for high‐risk elderly patients with low BMI, hypoalbuminemia, or elevated D‐dimer. Among the 42 patients who ultimately developed RFS, those receiving routine combined supplementation at the initiation of nutritional support achieved markedly improved clinical outcomes compared with unsupplemented counterparts. Specifically, supplemented RFS patients displayed a 13.4‐day shorter average hospital stay, lower in‐hospital mortality (16.7% vs. 43.3%), higher 28‐day (75.0% vs. 36.7%) and 90‐day survival rates (50.0% vs. 16.7%), and reduced incidence of respiratory and cardiac insufficiency and shortened duration of mechanical ventilation, pressure ulcer management, and systemic antibiotic therapy (all *p* < 0.05). Only liver, renal, coagulation, and multiple organ dysfunction showed no intergroup differences between the two RFS subgroups.

Contrary to concerns that parenteral nutrition might increase electrolyte disturbances, feeding delivery route (enteral/parenteral regardless of administration method/combined) had no impact on RFS risk.

### 4.2. Prognostic Impact of RFS and Hypophosphatemia

RFS patients experienced markedly prolonged hospital stay, reduced short‐ and long‐term survival, and higher risks of respiratory and cardiac dysfunction. They also required longer mechanical ventilation and systemic antibiotic treatment compared with non‐RFS counterparts. These findings align with Bioletto F et al.’s [5] meta‐analysis, which linked RFS to increased medium‐term mortality (≤ 6 months) via persistent metabolic dysfunction. Critically, hypophosphatemia (a hallmark of RFS) further worsened the prognosis: within the RFS group, hypophosphatemic patients had 2.4x higher mortality (54.5% vs. 25.0%) and 66% lower 90‐day survival (15.4% vs. 51.7%) than those with normal phosphate levels. The mechanisms underlying hypophosphatemia’s prognostic impact are well‐supported by physiology: Phosphate is essential for ATP synthesis, cell membrane integrity, and oxygen transport (via 2,3‐diphosphoglycerate in red blood cells) [33, 34]. Low phosphate reduces ATP production, impairing respiratory muscle function (prolonging ventilation) and myocardial contractility (increasing heart failure risk). It also shifts the oxygen dissociation curve leftward, reducing tissue oxygen delivery and exacerbating organ ischemia, explaining the higher rates of pressure ulcers and infection in hypophosphatemic patients.

### 4.3. Feeding Delivery Route and Stepwise Nutritional Therapy

Contrary to concerns that parenteral nutrition exacerbates electrolyte derangements, feeding delivery route—including enteral, parenteral (irrespective of vascular access type), and combined nutrition—exerted no independent effect on RFS risk. Stepwise nutritional therapy yielded superior clinical outcomes compared with fixed standard‐energy feeding: it shortened hospital length of stay, elevated 28‐day and 90‐day survival rates, and lowered the risk of respiratory and cardiac complications. These findings align with NICE [6] and ESPEN guidelines recommendations [25, 26], which advocate gradual energy up‐titration to blunt the catabolism‐to‐anabolism shift driving electrolyte depletion. When paired with targeted rescue electrolyte repletion (e.g., IV phosphate for serum phosphate ≤ 0.6 mmol/L), stepwise refeeding produced synergistic benefits that attenuated RFS severity. Serial electrolyte monitoring validated this combined strategy: serum phosphate, magnesium, and potassium concentrations declined markedly by Day 3 of nutrition initiation in patients with RFS (*p* < 0.05) but recovered to baseline by Day 7, consistent with the typical 72–96‐h window for RFS onset and affirming the value of early monitoring and intervention.

It is noteworthy that the application of calorie restriction for RFS prevention and management varies across patient populations. For critically ill and elderly malnourished adults, multiple high‐quality studies including landmark randomized controlled trials have demonstrated that moderate calorie restriction and stepwise refeeding can reduce electrolyte disturbances and RFS‐related organ damage without increasing adverse risks. Doig GS et al.’s multicenter RCT [22] confirmed that restricted caloric intake improved long‐term survival in critically ill patients who developed RFS, and relevant meta‐analyses also supported the safety of permissive underfeeding during the acute phase of critical illness.

In contrast, clinical guidelines for children and adults with eating disorders (e.g., anorexia nervosa) explicitly discourage excessive calorie restriction [36, 37]. In this population, overly low nutritional intake may lead to underfeeding syndrome and worsen malnutrition, which poses greater threats than RFS itself. The discrepancy stems from differences in underlying diseases, age, physiological reserve, and primary causes of malnutrition. For our study population (elderly hospitalized patients with chronic diseases and acute critical conditions), individualized moderate calorie restriction combined with routine supplementation of phosphate, magnesium, potassium, and vitamin B1 remains a safe and effective strategy to control RFS severity.

In clinical practice, IV phosphate supplementation with dextrose carries potential risks for severely malnourished elderly patients. Dextrose infusion can stimulate insulin secretion, which accelerates phosphate translocation from extracellular fluid into cells and worsens hypophosphatemia. Meanwhile, insulin may alter renal tubular reabsorption and increase fluid retention, leading to edema, which is superimposed on hypoalbuminemia‐related edema. Referring to clinical experiences in patients with eating disorders, we adopted optimized replacement strategies in this cohort: We limited the use of dextrose‐containing diluents, prioritized normal saline for IV preparations, and selected oral phosphate supplements whenever feasible. This protocol effectively reduced the risk of aggravated hypophosphatemia and fluid overload during correction.

### 4.4. Clinical Manifestations and Diagnostic Challenges

RFS manifestations in this cohort were predominantly respiratory (40.5%) and circulatory (35.7%)—mirroring Friedli N et al.’s [19] 2018 findings. Respiratory symptoms (e.g., tachypnea and respiratory failure) stemmed from hypophosphatemic respiratory muscle weakness, while circulatory symptoms (e.g., arrhythmia and heart failure) resulted from hypomagnesemia/hypokalemia and thiamine deficiency. However, these symptoms are often nonspecific and overlap with underlying comorbidities (e.g., COPD and heart failure) common in the elderly, contributing to missed diagnoses. For example, 48% of RFS patients had tumors, and 17% had pneumonia; their baseline respiratory/cardiac impairment may have masked RFS‐related deterioration. This highlights the need for proactive monitoring (e.g., daily electrolyte checks for 72 h postfeeding) in high‐risk patients, rather than relying on clinical symptoms alone.

### 4.5. Limitations and Future Directions

This study has several limitations. First, its retrospective design may introduce selection bias (e.g., incomplete data on preadmission nutritional status). Second, the small sample size (*n* = 93) limits the generalizability of the findings, particularly for subgroups (e.g., patients with rare comorbidities). Third, RFS diagnosis relied on Friedli N et al.’s [10] criteria (phosphate drop > 30% or < 0.6 mmol/L plus electrolyte disturbances), which lack universal consensus; future studies should validate these criteria against standardized endpoints (e.g., organ failure). Future research should focus on: (1) developing an RFS risk prediction model incorporating BMI, albumin, D‐dimer, and comorbidities (e.g., tumors and infection); (2) evaluating long‐term outcomes (e.g., 1‐year survival) of stepwise therapy; and (3) testing novel biomarkers (e.g., IGF‐1 [7]) to improve early RFS detection.

### 4.6. Clinical Implications

For clinical practice, this study reinforces three key strategies: (1) Risk Stratification: Screen elderly malnourished patients for low BMI (< 18 kg/m^2^), low albumin (< 30 g/L), and high D‐dimer (> 5 μg/mL) to identify RFS risk. (2) Proactive Prevention: In geriatric inpatients who have even seemingly low risk for RFS, administer bundled phosphate, magnesium, potassium, and vitamin B1 supplementation upon hospital admission using the standardized daily doses applied in this cohort: thiamine 200–300 mg daily, phosphate 0.3–0.6 mmol/kg daily, magnesium 0.2 mmol/kg intravenously or 0.4 mmol/kg orally daily, and potassium 2–4 mmol/kg daily. Additionally, for the first 10–14 days of refeeding in those at higher risk for RFS, implement energy‐restricted therapy (10–29 kcal/kg/day) before gradual energy escalation to standard caloric targets. (3) Targeted Management: Initiate stepwise nutritional therapy in RFS patients, with daily electrolyte monitoring for 72 h and prompt correction of hypophosphatemia. Timely electrolyte correction was defined as daily monitoring of serum phosphate, magnesium, and potassium within the first 72 h after nutrition initiation, plus immediate supplementation once abnormalities were detected. Its efficacy was evaluated by hospital length of stay, 28‐day and 90‐day survival rates, and organ dysfunction incidence. By implementing these strategies, clinicians can reduce RFS incidence and improve outcomes in this high‐risk population.

In conclusion, low BMI, low serum albumin, and elevated D‐dimer are key risk factors for RFS in malnourished elderly patients. RFS acts as an important risk marker for poor clinical outcomes, especially when accompanied by hypophosphatemia. Short‐term restricted energy feeding was adopted in the acute phase to reduce RFS risk. Stepwise nutritional therapy and routine provision of electrolyte and vitamin B1 supplementation effectively mitigate these risks, highlighting the need for standardized, evidence‐based RFS management in geriatric clinical practice.

## Funding

This study was supported by the Jiangsu Commission of Health (LKZ2023007), Soochow University (SDFEYGZ2219, GZK12023018), and Jiangsu Provincial Department of Science and Technology (BE2023704).

## Conflicts of Interest

The authors declare no conflicts of interest.

## Supporting Information

Additional supporting information can be found online in the Supporting Information section.

## Supporting information


**Supporting Information** Supporting Table 1: Cross‐distribution of patients stratified by feeding route, prophylactic multimicronutrient supporting, and initial energy regimen across RFS and non‐RFS groups and presents sample sizes for all nutritional intervention subgroups. Supporting Table 2: Baseline nutritional risk stratification of patients who received standardized phosphate–magnesium–potassium–thiamine micronutrient prophylaxis, categorizing eligible high‐malnutrition‐risk patients versus low‐risk patients without formal supplementation indications. Supporting Table 3: Adjusted binary logistic regression model accounting for baseline supporting eligibility, isolating the independent protective effect of combined micronutrient prophylaxis against RFS. Supporting Table 4: Subgroup comparison of RFS incidence among parenteral nutrition recipients stratified by vascular access type (PICC, medium long‐term central catheter, infusion port, and deep venous catheter). Supporting Table 5: Stratified prognostic comparison within the RFS cohort (*n* =  42): clinical outcomes, organ dysfunction rates, and duration of supportive care between patients who received upfront multimicronutrient supplementation and unsupplemented patients. Supporting Table 6: Multivariate binary logistic regression analysis identifying independent baseline laboratory and anthropometric predictors of RFS (BMI, serum albumin, and D‐dimer). Supporting Table 7: Quantification of adherence to institutional micronutrient supplementation guidelines: proportion of eligible high‐risk patients who received prophylactic electrolyte‐vitamin B1 bundles, plus risk distribution of all supported individuals. Supporting Table 8: Fully adjusted logistic regression model controlling for baseline clinical repletion eligibility to confirm the protective impact of combined micronutrient prophylaxis independent of preexisting malnutrition severity. Supporting Table 9: Full comparative prognostic indicators between RFS and non‐RFS groups, including hospital length of stay, mortality, survival rates, organ dysfunction incidence, and cumulative days of mechanical ventilation, pressure ulcer management, and systemic antibiotic therapy. Supporting Table 10: Serial dynamic serum phosphate, magnesium, and potassium concentrations at baseline, Day 3, and Day 7 after initiation of nutritional support for both RFS and non‐RFS patient groups. Supporting Figure 1: Pie chart illustrating the primary admission disease distribution among patients diagnosed with RFS (tumors, pneumonia, digestive inflammatory disorders, and other comorbidities).

## Data Availability

The datasets used and/or analyzed during the current study are available from the corresponding author on reasonable request.
